# The development and validation of the Discrimination and Stigma Scale Ultra Short for People Living with Dementia (DISCUS-Dementia)

**DOI:** 10.1192/bjo.2023.551

**Published:** 2023-08-31

**Authors:** Jem Bhatt, Elaine Brohan, Drew Blasco, Déborah Oliveira, Ioannis Bakolis, Adelina Comas-Herrera, Francesco D'Amico, Nicolas Farina, Martin Knapp, Madeleine Stevens, Graham Thornicroft, Emma Wilson, Maximilian Salcher-Konrad, Lawrence H. Yang, Sara Evans-Lacko

**Affiliations:** UCL Unit for Stigma Research, Research Department of Clinical, Educational and Health Psychology, University College London, UK; Centre for Global Mental Health, Institute of Psychiatry, Psychology and Neuroscience, King's College London, UK; Department of Health Behavior and Health Education, School of Public Health, University of Michigan, Michigan, USA; Department of Social and Behavioral Sciences, School of Global Public Health, New York University, New York, USA; and Department of Social and Behavioral Health, School of Public Health, University of Nevada, Las Vegas, USA; Faculty of Nursing, Universidad Andrés Bello, Campus Vina del Mar,Chile; and Millennium Institute for Care Research (MICARE), Santiago,Chile; Department of Biostatistics and Health Informatics, Institute of Psychiatry, Psychology and Neuroscience, King's College London, UK; Care Policy and Evaluation Centre, London School of Economics and Political Science, UK; Centre for Dementia Studies, Brighton and Sussex Medical School, UK; Centre for Global Mental Health and Centre for Implementation Science, Health Service and Population Research Department, Institute of Psychiatry, Psychology and Neuroscience, King's College London, UK; ESRC Centre for Society and Mental Health, King's College London, UK; WHO Collaborating Centre for Pharmaceutical Pricing and Reimbursement Policies, Pharmacoeconomics Department, Austrian National Public Health Institute (Gesundheit Österreich GmbH/GÖG), Austria; Department of Epidemiology, Mailman School of Public Health, Columbia University, New York, USA

**Keywords:** Stigma and discrimination, epidemiology, low- and middle-income countries, rating scales, dementias/neurodegenerative diseases

## Abstract

**Background:**

The recent World Health Organization (WHO) blueprint for dementia research and Lancet Commission on ending stigma and discrimination in mental health has identified a gap around dementia-related measures of stigma and discrimination that can be used in different cultural, language and regional contexts.

**Aims:**

We aimed to characterise experiences of discrimination, and report initial psychometric properties of a new tool to capture these experiences, among a global sample of people living with dementia.

**Method:**

We analysed data from 704 people living with dementia who took part in a global survey from 33 different countries and territories. Psychometric properties were examined, including internal consistency and construct validity.

**Results:**

A total of 83% of participants reported discrimination in one or more areas of life, and this was similar across WHO Regions. The exploratory factor analysis factor loadings and scree plot supported a unidimensional structure for the Discrimination and Stigma Scale Ultra Short for People Living with Dementia (DISCUS-Dementia). The instrument demonstrated excellent internal consistency, with most of the construct validity hypotheses being confirmed and qualitative responses demonstrating face validity.

**Conclusions:**

Our analyses suggest that the DISCUS-Dementia performs well with a global sample of people living with dementia. This scale can be integrated into large-scale studies to understand factors associated with stigma and discrimination. It can also provide an opportunity for a structured discussion around stigma and discrimination experiences important to people living with dementia, as well as planning psychosocial services and initiatives to reduce stigma and discrimination.

There are approximately 52.2 million people living with dementia globally, and it has been estimated that this number will increase to 150 million by 2050.^1^ The global cost of dementia is approximately $1.3 trillion, which is estimated to increase to $2.8 trillion by 2030.^2^ People living with dementia have to manage clinical symptoms and disability, but they also face stigma and discrimination, which can undermine life goals, reduce participation in meaningful activities and lower well-being and quality of life.^3,4^ Stigma can be conceptualised as comprising problems of knowledge (ignorance), attitudes (prejudice) and behaviour (discrimination).^4^ Discrimination is considered the behavioural enactment of ignorance and prejudice. The consequences of stigma are often described by people with mental health conditions as being worse than the condition itself.^4^ However, most research focuses on clinical and care experiences of dementia rather than social consequences, and is often conducted about or for people living with dementia, rather than with them.^5^ Both qualitative and quantitative evidence on experiences of discrimination among people living with dementia is lacking.^6^ This is particularly true in low- and middle-income countries (LMICs), where nearly 70% of people currently living with dementia reside.^7^

Previous dementia-related stigma research has mostly focused on quantifying stereotypes and prejudice toward people living with dementia from family members or the general public.^6,8^ Capturing such ‘public stigma’ has value, but is limited by relying on views of potential ‘stigmatisers’ rather than people living with dementia, potentially increasing social desirability bias and underestimating the scale of the problem. A further limitation is that studies investigating public stigma often omit the context in which stigmatisation occurred (e.g. hospital settings, social situations), as well as the consequence (e.g. excluded from social gatherings). Some research has explored the experience of stigma and discrimination among people living with dementia and their care partners by using qualitative methods, including participants from LMICs,^9–12^ and a few studies have quantitatively assessed self-reported experiences of people living with dementia by using the Stigma Impact Scale (SIS) and Stigma Experience Scale.^13,14^ These measures, however, demonstrate low reliability for people living with dementia, given they were not designed for this population and were validated among small samples in Western high-income countries. Additionally, these measures focus on perceived stigma (e.g. ‘I have worried’) rather than experiences of discrimination. A standardised tool to assess dementia-related stigma and discrimination across varying global contexts would be of great value. The Discrimination and Stigma Scale Ultra Short (DISCUS) is an existing tool that assesses the scope and context of experienced discrimination among people with mental health conditions, and has previously demonstrated excellent reliability and factor structure in large global samples.^15,16^ We sought to adapt the DISCUS for people living with dementia, to address the aforementioned research gaps and test its psychometric properties. We refer to the adapted instrument as the Discrimination and Stigma Scale Ultra Short for People Living with Dementia (DISCUS-Dementia).

## Overarching aim

We aimed to characterise the experiences and frequency of discrimination among a global sample of people living with dementia, as well as describe the psychometric properties of a new tool to capture these experiences.

## Research objectives

The research objectives for this study were to adapt the DISCUS for use among people living with dementia and describe the measure and its psychometric properties across and within World Health Organization (WHO) regions; to describe and compare frequency of endorsement of discrimination by life domains among people living with dementia, assessed using the DISCUS-Dementia, overall and by WHO Region; and to describe examples of discrimination reported by people living with dementia according to different life domains.

## Method

### Study design

Data were gathered as part of a large, cross-sectional global survey for the World Alzheimer Report 2019, which surveyed the general public, healthcare professionals, caregivers and people living with dementia.^3^ Here, we focus on data from participants who self-identified as living with dementia.

### Participants

We partnered with Alzheimer's Disease International (ADI) to recruit participants. We ran webinars (in English and Spanish) for members of ADI organisations to discuss recruitment strategies. Participants were also recruited from various online platforms: dementia discussion boards, social media, the ADI website, mailing lists of national Alzheimer's or similar associations, social care organisations, support groups and other third-sector or faith-based organisations. Written informed consent was obtained from all patients.

To achieve good representation, outreach by health and community workers in rural regions and places without internet access was done by hardcopy forms; ADI member organisations facilitating internet access at their own offices and events; and use of Mobenzi for Windows (Mobenzi Technologies, Cape Town, South Africa; see https://www.engineeringforchange.org/solutions/product/mobenzi/), a tool supporting offline data collection.

People living with dementia (*n* = 742) from 33 countries responded to this survey (Supplementary Table 2 available at https://doi.org/10.1192/bjo.2023.551). Given the limited amount of respondents from the African, Eastern Mediterranean and South-East Asia Regions, our psychometric study sample included 704 people living with dementia from three WHO Regions – the European Region, Region of the Americas and Western Pacific Region – representing 33 countries and/or territories worldwide. Participant sociodemographic characteristics, overall and by WHO Region, are presented in Supplementary Table 2. Most participants completed the survey alone (86.2%), whereas others required support (5.3%) and 8.5% did not respond to this question.

The authors assert that all procedures contributing to this work comply with the ethical standards of the relevant national and institutional committees on human experimentation and with the Helsinki Declaration of 1975, as revised in 2008. As we analysed fully anonymised data collected by an outside organisation (ADI), all procedures involving human patients were approved by the London School of Economics and Political Science self-certification process (reference: CPEC-LSE-2019-SE-06).

### Development, translation and final version of the DISCUS-Dementia

We adapted the original DISCUS, globally validated for people with mental illness, which collects quantitative information on experience of discrimination across 11 life areas.^16^ Those who report discrimination are then asked to provide qualitative examples. As the original measure focused on mental illness, we first reviewed DISCUS items to ensure domains were relevant and sufficient for people living with dementia. We identified studies from a recent systematic review that captured the global literature on stigma and dementia, and extracted constructs from primary studies included in it.^6^ We then systematically mapped identified constructs and compared them with items included in the DISCUS, to identify overlap and potential gaps. We further validated constructs with stakeholders (including people living with dementia, experts in stigma and dementia-related research and representatives from ADI associations around the world, to include perspectives from a range of cultures and contexts). As a result of this process, three items were added, to form the DISCUS-Dementia (Supplementary Table 1). To further improve accessibility, we translated the survey into 32 languages. All translations adhered to WHO guidelines^17^ and were mostly done by staff of ADI member organisations.

The DISCUS-Dementia comprised 14 items covering domains of everyday life, including privacy, personal safety, responsibilities and rights, social, familial and intimate relationships, housing and healthcare. Participants rated items on a four-point scale (0 being ‘not applicable’, 1 being ‘a little’, 2 being ‘moderately’ and 3 being ‘a lot’). Each item had a free-text option to include examples of discrimination.

### Additional measures

#### Warwick-Edinburgh Mental Well-Being Scale

The Warwick-Edinburgh Mental Well-Being Scale (WEMWBS) is a 14-item scale that assesses mental well-being.^18^ Although not dementia specific, it has been used to assess mental well-being among people living with dementia in several studies,^19^ and has been extensively validated in adult populations with good psychometric properties (e.g. internal consistency Cronbach's alpha of 0.91–0.94; test–retest reliability intraclass correlation coefficient of 0.83).^20^ Scores range from 14 to 70, with higher scores indicating greater positive mental well-being.

#### Dementia Quality of Life Instrument

The Dementia Quality of Life Instrument (DQoL) is a disease-specific measure of quality of life, designed to be self-completed by persons with mild-to-moderate dementia.^21^ Previous research in three European countries found the DQoL to be negatively associated with stigma impact (assessed by the SIS).^22^ Based on feedback from our advisory panel of stigma experts and people with lived experience, we included three of the five subscales that were significantly associated with the SIS: self-esteem, negative affect and feelings of belonging.^22^ Previous research suggests these subscales have demonstrated moderate-to-good internal consistency (Cronbach's alpha of 0.67–0.89) and test–retest reliability (intraclass correlation coefficient of 0.64–0.74).^21^ Higher scores indicate more positive quality of life.

#### SIS

The SIS is a 21-item measure rated on a Likert scale from 1 to 4, with the addition of 0 for items that participants felt were ‘not applicable’. As a result of the development and adaptation process and stakeholder feedback,^3^ one item was deemed irrelevant and removed (item 21: ‘Changes in my appearance have affected my social life’). In this case, the total score ranged from 20 to 92, with higher scores indicating greater perceived stigma impact. In a study of people living with dementia, it showed good internal consistency (Cronbach's alpha of 0.906) and test–retest reliability (intraclass correlation coefficient of 0.774).^23^

#### Sociodemographic characteristics

To characterise our sample, we collected information on gender, age, highest level of education, country/territory of residence, urbanicity and employment status.

### Data analysis

Survey weights were developed to match characteristics of the sample to the nationally representative characteristics in each country, according to gender, age and education. Responses with weights >20 were excluded (<0.2% of the sample) because of non-representativeness.

### Face validity and concept checking

Qualitative responses were assessed for respondent understanding (face validity) based on whether the response was relevant to the item's focus (e.g. ‘Have you been treated unfairly in housing?’). A simple coding scheme was applied to indicate the relevance of each response (yes/no) and the reason for any irrelevance. Initially, 10% of responses were coded independently by three researchers (J.B., D.B. and D.O.). Discrepancies were discussed until agreement was reached. Remaining responses were coded individually. Coders met to discuss responses that were unclear, and to reach consensus. Exemplar responses for each question were identified and included in Table 1. All qualitative examples provided by respondents were translated into English before any data were reviewed and checked for accuracy and consistency by the research team.

**Table 1:** DISCUS-Dementia face validity and exemplar quotes

Item	DISCUS-Dementia item	*n*^a^	Participant understanding (%)^b^	Exemplar quote
1	Have you been treated unfairly in your levels of privacy?	38	52.6	‘I know my health records have been shared without my consent … and I have also felt the need to share them to prevent public defamation of the possibility of me faking dementia.’ (Female, 60 years, Australia, Western Pacific Region)
2	Have you been treated unfairly in your personal safety and security?	41	85.4	‘Verbal abuse and some physical abuse from loved one.’ (Female, 65 years, USA, Region of the Americas)
3	Have you had rights or responsibilities unfairly taken away from you?	67	86.6	‘My wife handles all finances even though I think I am capable.’ (Male, 79 years, Canada, Region of the Americas)
4	Do people do things for you that you could do yourself because they know you have dementia?	154	97.4	‘The nurses in nursing home like to spoon feed me while I can eat unassisted.’ (Male, 76 years, Malaysia, Western Pacific Region) ‘A close friend sometimes finishes my sentence as I take time to find my words. I am in very early stage and aphasia is my major problem.’ (Female, 75 years, USA, Region of the Americas)
5	Have you been told that you couldn't do something that you still thought you could do?	172	91.9	‘I can't take grandchildren out, I can't do cooking alone, I can't control my finances, I make friends of people who my family say are unsafe, I can't travel as I get lost.’ (Male, 62 years, UK, European Region)
6	Do people often joke about your dementia symptoms?	118	97.5	‘If I stutter when talking to people who don't know it's one of the symptoms. Then they'll copy me for a second. A colleague regularly imitates me or says that I will soon forgot it anyway. She means well and I don't mind when she does it. Others sometimes feel it's not done and find it more embarrassing than I do. My husband and I also regularly joke about it. There is also self-mockery.’ (Female, 62 years, The Netherlands, European Region)
7	Because of your dementia have some people not take your opinions seriously?	134	94.8	‘I will express my opinion and they either stay quiet or interrupt me before I can finish my thoughts.’ (Female, 63 years, USA, Region of the Americas)
8	Have you been avoided or shunned by people who know that you have dementia?	92	100.0	‘I outed as soon as I was diagnosed. I was seriously SHUNNED. My busy social life disappeared and my phone stopped ringing. I felt like disappearing to where nobody could compare me to pre-personality.’ (Female, 66 years, Australia, Western Pacific Region)
9	Have you been treated unfairly in making or keeping friends?	113	95.6	‘A friend thought I could not cope with things and felt I should not be out of the house. We had words over it and we really haven't spoken much since. I think I have found my true friends and that's all I need.’ (Male, 56 years, UK, European Region)
10	Have you been treated unfairly in your social life?	85	95.3	‘I was not able to attend an event because I didn't have a caregiver to accompany me. I live by myself and don't need one, yet.’ (Female, 65 years, USA, Region of the Americas) ‘I used to be active in a club, but now I am a non-person.’ (Male, age not provided, USA, Region of the Americas)
11	Have you been treated unfairly in dating or intimate relationships?	86	96.5	‘I haven't dated in years. Had my confidence blown out of the water towards this. I met a nurse who ironically worked at a care facility for people with dementia. On our second date I mentioned I had Alzheimer's. Thought I should get that out of the way. She said I have met some losers in my time but you take the cake.’ (Male, 61 years, Canada, Region of the Americas)
12	Have you been treated unfairly by your children or other family members?	111	97.3	‘I have a daughter who has distanced her and her family since I was diagnosed.’ (Female, 55 years, UK, European Region)
13	Have you been treated unfairly by health or medical staff?	72	90.3	‘Primary care doctor said to my face, “too bad that euthanasia is illegal here”. Psychologist fired me after 9 years as his patient. Neurologist is dismissive.’ (Female, 65 years, USA, Region of the Americas) ‘I've been addressed and treated like a toddler several times. Very painful and belittling. I have also been blamed for something I had absolutely no part in. When explaining things and during conversations they also don't take the limitations of dementia at a young age into account at all. And when you ask people to, for example, slow down or use shorter sentences because information processing is a bit tricky, people get annoyed or start talking as well-articulated simpletons.’ (Female, 55 years, The Netherlands, European Region)
14	Have you been treated unfairly in housing? (including having to move your house)	28	92.9	‘Last summer I was brought to a crisis shelter while I thought I was going on a little holiday.’ (Female, 79 years, The Netherlands, European Region)

DISC-Dementia, Discrimination and Stigma Scale Ultra Short for People Living with Dementia.

a. Number of text responses recorded for DISC-Dementia item.

b. Percentage of qualitative responses that were coded by authors as satisfactorily reflecting participant understanding of the item.

### Psychometric analysis

Summary statistics were calculated for all measures. DISCUS-Dementia scores were calculated for each item and for the total scale, and then summarised for each WHO Region with sufficient sample size. We excluded participants from the African, South-East Asia and Eastern Mediterranean Regions because of low sample size (Supplementary Table 2). For reference, data from participants in these regions are described in Supplementary Table 2. DISCUS-Dementia item means were also calculated (range 0–3; 0 being ‘not at all’, 1 being ‘a little’, 2 being ‘moderately’ and 3 being ‘a lot’). ‘Not applicable’ answers were recoded as ‘0’ for analyses with the total sample, as well as by each included WHO Region.

Psychometric analysis focused on (a) examining scaling assumptions in a dementia population, (b) evaluating scale reliability and (c) evaluating construct validity.

When more than two item scores were missing, the mean score was not calculated (in 33 out of 704 cases); otherwise, subscale and total scores were generated in keeping with scoring instructions for each instrument. Analysis was performed with SPSS version 26 for Windows and Stata version 16 for Windows.

#### DISCUS-Dementia scaling assumptions

Exploratory factor analysis (EFA) was used to test the assumption of unidimensionality, which has been established for other DISCUS populations (e.g. people with depression or schizophrenia).^16,24^ Varimax rotation was applied to improve interpretability of factors obtained. Eigenvalues, scree plot and proportion of variance explained by each factor were used to evaluate factor structure. Each item having a loading of ≥0.4 on one factor, with a lower loading on other factors, was considered as a threshold for unidimensionality.^25^

As a sensitivity analysis, EFA was also run separately for each region.

#### Reliability and validity

Reliability of the DISCUS-Dementia was assessed with Cronbach's alpha, with a criterion of α ≥ 0.70 indicative of appropriate internal consistency; α > 0.90 were also flagged, as this may indicate item redundancy. Two aspects of construct validity were assessed: known-groups method and convergent validity.^16^ The known-groups method assessed differences in scores between participants who differed on identified clinical variables. The criterion was considered met when significantly different DISCUS scores (defined as *P*<0.05) were obtained between sample subgroups. The following groups were considered: WEMWBS score (higher mental well-being (score of ≥42) versus lower mental well-being (score of ≤41)) and DQoL score (higher quality of life (median score of >2.25) versus lower quality of life (median score of ≤2.25)). In convergent validity analyses, it was hypothesised there would be moderate positive correlation (*r* = 0.30–0.49) between mean DISCUS-Dementia score and SIS.^26^

As a sensitivity analysis, psychometric properties were analysed for the subgroup of individuals who required support to complete the survey. This subgroup was considered to reflect those who may experience greater impairment owing to dementia symptoms. Significance tests are not included for this analysis because of small sample size.

## Results

Most DISCUS items demonstrated face validity, with almost all qualitative responses reflecting participants understood the item (≥85%), with the exception of one item (‘Have you experienced unfair treatment in your levels of privacy?’; 52.6%; see Table 1).

### Psychometric properties of the DISCUS-Dementia

The EFA factor loading and scree plot supported a unidimensional structure (Table 2 and Supplementary Fig. 1). All items loaded most highly on the first factor, with loadings on the second factor lower in all cases (<0.3). The percent of total variance explained by the first factor was 52.4%, with an additional 7.4% explained with inclusion of a second factor. Sensitivity analysis suggested this model was also appropriate in each regional subsample, with 48.8% of variance explained by the first factor in the Region of the Americas, 55.1% in the European Region and 51.0% in the Western Pacific Region. The interpretability of each model was not improved by inclusion of a second factor.

**Table 2:** Exploratory factor analysis

Item	Factor 1	Factor 2
Have you been treated unfairly in making or keeping friends?	0.632	0.252
Have you been treated unfairly in dating or intimate relationships?	0.472	0.244
Have you been treated unfairly in housing? (Including having to move your house)	0.478	0.111
Have you been treated unfairly by your children or other family members?	0.681	0.198
Have you had rights or responsibilities unfairly taken away from you?	0.708	0.165
Have you been told that you couldn't do something that you still thought you could do?	0.676	0.194
Have you been treated unfairly in your social life?	0.795	0.162
Because of your dementia have some people not taken your opinions seriously?	0.683	0.215
Do people often joke about your dementia symptoms?	0.726	0.077
Have you been treated unfairly in your levels of privacy?	0.760	−0.206
Have you been treated unfairly in your personal safety and security?	0.845	−0.324
Have you been treated unfairly by health or medical staff?	0.797	−0.227
Do people do things for you that you could do yourself because they know you have dementia?	0.703	0.045
Have you been avoided or shunned by people who know that you have dementia?	0.753	0.056

Reliability of DISCUS-Dementia items and total score was satisfactorily established. Cronbach's alpha coefficient was excellent for all regions (α = 0.90–0.93), and only negligible increases (0.01) were noted in two of the three regions when an item was dropped (Table 3).

**Table 3:** Summary of psychometric properties of the DISCUS-Dementia by region

Property	Subcomponent			European Region (*n* = 333)	Region of the Americas (*n* = 260)	Western Pacific Region (*n* = 111)	Total (*N* = 704)
Scaling assumptions^a^	Inter-item polychoric correlations (mean, range)			Mean: 0.58, 0.30–0.82	Mean: 0.50, 0.13–0.84	Mean: 0.54, 0.30–0.82	Mean: 0.57, 0.29–0.83
	Corrected item-total correlations			0.53–0.80	0.40–0.74	0.47–0.76	0.48–0.77
Reliability	Internal consistency			α = 0.93	α = 0.90	α = 0.92	α = 0.92
	Changes with item deletion			Minor increase with removal of item 3 ‘housing’ α = 0.94	Minor increase with removal of item 2 ‘intimate relationships’ α = 0.91	Does not improve with removal	Minor increase with removal of item 3 ‘housing’ α = 0.93
Construct validity	Convergent validity^b^	Stigma Impact Scale		0.039	0.30*	0.58*	0.30*
	Known-groups method^c^ (Wilcoxon rank-sum)	WEMWBS	Higher mental well-being ≥42	0.21*	0.21*	0.21*	0.21*
			Lower mental well-being <42	0.43	0.43	0.45	0.43
		DQoL	Higher QoL (> median 2.25)	0.14*	0.13*	0.14*	0.14*
			Lower QoL (≤ median 2.25)	0.29	0.36	0.43	0.36

DISCUS-Dementia, Discrimination and Stigma Scale Ultra Short for People Living with Dementia; WEMWBS, Warwick-Edinburgh Mental Well-Being Scale; DQoL, Dementia Quality of Life Instrument; QoL, quality of life.

a. See Table 3 for results of exploratory factor analysis.

b. Spearman's rho was used for convergent validity analysis because of the non-normality of DISCUS-Dementia mean scores.

c. Wilcoxon rank-sum test was used to compare median scores for known-groups analysis because of the non-normality of DISCUS-Dementia mean scores.

*P < 0.001.

There was evidence of convergent validity in the total sample and in the Region of the Americas and Western Pacific Region, as moderate/high correlation was seen between mean DISCUS score and SIS. Convergent validity was not supported in the European Region. There was evidence of construct validity using the known-groups method in the total sample and in the Region of the Americas, European Region and Western Pacific Region, with a significant difference between groups seen for each hypothesised relationship (mental well-being and quality of life). There was initial evidence that the psychometric properties, including scaling assumptions, reliability and validity, were replicated in the subgroup of individuals who required support to complete the survey, with inter-item polychoric correlation (mean 0.57), internal consistency (α = 0.93), convergent validity (0.33), known-group method WEMWBS (0.14 higher mental well-being *v*. 0.45 lower mental well-being) and known-group method DQoL (0.33 higher quality of life *v*. 0.40 lower quality of life).

### Prevalence of discrimination

Overall, results indicated that when examining discrimination by life domain across all three regions included in analysis, the most highly endorsed DISCUS-Dementia items (i.e. respondents choosing either ‘a little’, ‘moderately’ or ‘a lot’) were: ‘Do people do things for you that you could do yourself because they know you have dementia?’ (51.3%), ‘Because of your dementia have some people not taken your opinions seriously?’ (50.2%) and ‘Have you been told that you couldn't do something that you still thought you could do?’ (44.0%) (Table 4).

**Table 4:** Endorsement of unfair treatment among people living with dementia overall and by region

DISC-Dementia item	European Region (*n* = 333)		Region of the Americas (*n* = 260)		Western Pacific Region (*n* = 111)		Total (*N* = 704)	
	Total	*n* (%)	Total	*n* (%)	Total	*n* (%)	Total	*n* (%)
Do people do things for you that you could do yourself because they know you have dementia?	322	160 (49.69)	258	140 (54.27)	105	51 (48.57)	685	351 (51.25)
Because of your dementia have some people not taken your opinions seriously?	321	139 (43.30)	256	146 (57.03)	106	58 (54.72)	683	343 (50.22)
Have you been told that you couldn't do something that you still thought you could do?	320	136 (42.50)	257	108 (42.02)	107	57 (53.27)	684	301 (44.01)
Have you been treated unfairly in making or keeping friends?	329	110 (33.44)	257	101 (39.30)	107	50 (46.73)	693	261 (37.67)
Do people often joke about your dementia symptoms?	322	102 (31.68)	259	84 (32.48)	105	39 (37.14)	686	225 (32.80)
Have you been avoided or shunned by people who know that you have dementia?	321	84 (26.17)	257	83 (32.29)	105	32 (30.47)	683	199 (29.13)
Have you been treated unfairly by your children or other family members?	327	69 (21.10)	259	90 (34.74)	109	38 (34.86)	695	197 (28.34)
Have you been treated unfairly in your social life?	320	67 (20.93)	255	82 (32.16)	106	33 (31.13)	681	182 (26.72)
Have you been treated unfairly in dating or intimate relationships?	324	74 (22.84)	257	59 (22.96)	108	38 (34.89)	689	170 (24.67)
Have you had rights or responsibilities unfairly taken away from you?	326	60 (18.40)	258	73 (28.29)	108	32 (29.63)	692	165 (23.85)
Have you been treated unfairly by health or medical staff?	320	53 (16.56)	259	51 (19.69)	106	23 (21.70)	685	127 (18.55)
Have you been treated unfairly in your levels of privacy?	317	44 (13.88)	259	26 (10.04)	105	22 (20.96)	681	92 (13.51)
Have you been treated unfairly in your personal safety and security?	321	27 (8.41)	257	40 (15.57)	105	24 (22.86)	683	91 (13.32)
Have you been treated unfairly in housing? (Including having to move your house)	323	24 (7.43)	259	23 (8.88)	104	17 (16.34)	686	64 (9.33)
Number of respondents who experienced at least one item	317	266 (85)	255	211 (82.80)	104	86 (82.90)	681	563 (82.80)

DISCUS-Dementia, Discrimination and Stigma Scale Ultra Short for People Living with Dementia.

The least commonly endorsed items were around privacy (13.5%), personal safety and security (13.3%) and housing (9.3%). When comparing prevalence of discrimination across WHO Regions, there was good alignment regarding the most and least commonly endorsed items. Overall, 82.8% of participants reported discrimination in one or more areas of life: 82.8% in the Region of the Americas, 85.0% in the European Region and 82.9% in the Western Pacific Region.

## Discussion

This paper describes the development and psychometric validation of the DISCUS-Dementia, which can be used to assess the nature, type and degree of experiences of stigma and discrimination among people living with dementia. It addresses a gap flagged in the recent 2022 WHO blueprint for dementia research,^27^ for measures of stigma and discrimination that can be used in different cultural, language and regional contexts. Findings demonstrate strong psychometric properties and characterise experiences of stigma and discrimination of people living with dementia from three global regions. Qualitative responses, describing examples of discrimination, provide further support for comprehensibility and face validity among people living with dementia.

The DISCUS-Dementia demonstrated excellent reliability across three WHO Regions, and performed similarly or better compared with the original DISCUS. Construct validity was demonstrated with known-groups validity, established using groups defined by quality of life and well-being in each region. Convergent validity was demonstrated in all regions apart from the European Region. In the European Region, stigma impact scores were, on average, similar to other regions; however, DISCUS scores were lower, on average, than in other regions. All other proposed thresholds for psychometric analysis were met in the European Region, and all thresholds across the other regions were met.

The DISCUS-Dementia addresses previous research gaps, providing an alternative to existing scales such as the SIS and Stigma Experience Scale, which focus on perceived stigma rather than experiences of stigma and discrimination, with validation in much smaller samples.^13,14^ Moreover, adaptation of the DISCUS-Dementia was undertaken in consultation with experts with lived experience and stigma research and practice experts from diverse settings, to consider experiences, domains and language that may be particularly relevant for people living with dementia. In contrast to the SIS, which had varying internal consistency for subscales ranging from 0.58 to 0.82, the DISCUS-Dementia demonstrated excellent internal consistency, with alpha values ranging from 0.90 to 0.92 across three WHO Regions. The adaptation process for the DISCUS-Dementia, which included tailoring an existing measure so that it aligned with what is most valued by or ‘what matters most’ for the target population, is recommended to improve construct validity.^28^

### Frequency and type of discrimination reported across regions

Across the sample, 83% reported discrimination in one or more areas of life; this was similar across WHO Regions. The three most commonly endorsed items were: ‘Do people do things for you that you could do yourself because they know you have dementia?’ (52.3%), ‘Because of your dementia, have some people not taken your opinions seriously?’ (50.2%) and ‘Have you been told that you couldn't do something that you still thought you could do?’ (44.0%). The high endorsement of these items corresponds to previous findings showing that people living with dementia often experience their autonomy being restricted and personhood undervalued or dismissed.^5,29^ This reflects previous understandings of consequences of stigma for people with dementia, including qualitative studies in LMICs from the perspective of people living with dementia and caregivers.^9–12^

Some regional variation was seen in levels of endorsement for DISCUS-Dementia items. The qualitative work helps clarify potential reasons for this in terms of structural and cultural differences, which underpin expectations around privacy, social life and housing (e.g. policy changes that place greater emphasis on rights and protections of people living with dementia concerning housing, benefits and social participation).^30^ This may have led to social change focused on altering disabling environments and structural changes to accommodate people living with dementia. Respondents in the Western Pacific Region reported greater experiences of discrimination in housing compared with the European Region and Region of the Americas, perhaps because of the way housing is managed in the Western Pacific Region. For example, a recent study emphasised the need to reform housing practices in China to reduce disabling people living with dementia through discrimination (infantilising), which may explain why participants from the Western Pacific Region endorsed discrimination in housing more highly than others.^31,32^ Although culture likely contributes to regional differences,^8,^^33^ at this stage we can only hypothesise why these differences exist.

### Strengths and limitations

Recruiting a large online sample allowed us to validate the DISCUS-Dementia among a considerable sample of people living with dementia across 33 diverse countries. Data on experiences of dementia-related stigma and discrimination have never previously been collected on such a large scale. A limitation is that most participants were educated women living in high-income countries, similar to other survey-based studies. Although we worked together with local Alzheimer's associations to support people without internet access to complete the survey, vulnerable subgroups may be underrepresented; this would be important to address in future research, and for wider generalisability of findings. To the extent possible, we attempted to address non-response bias by weighting results to be representative within each country in relation to age, gender and education. Nevertheless, further work is needed to explore how the DISCUS-Dementia performs among, for example, individuals with less education and those who live in remote areas and in other geographic settings, particularly low-income countries. We did attempt to include participants without internet access, and our sample did include participants who only completed primary school education or less (5%) and who lived in rural (9%) or semi-rural (23%) areas. Nonetheless, such underrepresented groups should be a focus of future research as they are likely further disadvantaged in a range of other areas (poverty, ableism); for example, by feeling less entitled to receive adequate care or having lower expectations about social interactions.

Another limitation is that we did not have a formal measure of dementia severity. As dementia is a heterogeneous condition with varied symptoms and stages, these differences may influence individuals’ experiences and perceptions of discrimination. Moreover, people living with dementia in LMICs tend to receive a diagnosis late in the course of illness and so are more likely to have severe symptoms once diagnosed, posing additional barriers to survey participation. We relied on participant self-report, which may introduce some error or uncertainty in identification of participants. We also conducted a sensitivity analysis to consider those who used proxy support to complete the survey. We argue that it is important to give people with dementia a voice and an opportunity to express their views and feelings. The DISCUS-Dementia is designed to capture the self-reported experiences of discrimination by people with dementia, regardless of their cognitive status, and does not rely on specific episodic memory or factual recall. Future work should explore ways in which the DISCUS-Dementia can be used to quantify experiences of people living with advanced dementia, with careful thought to data collection methodology and accommodations for participants with varying levels of symptom severity.

### Research and practice implications

The DISCUS-Dementia is a reliable, valid and acceptable measure for assessing experiences of stigma and discrimination among people living with dementia. This scale can be integrated into large-scale studies to understand prevalence of stigma and discrimination experienced by people living with dementia, including how to reduce the effects of stigma and discrimination among people living with dementia, their caregivers and families.

It is beyond the scope of our study to present an in-depth ethnographic perspective on the way cultural differences in experience of stigma and discrimination may underpin findings. However, our qualitative and quantitative findings provide a basis for hypothesis generation, and future work may look to address this pertinent question. Finally, this measure also has clinical relevance. Given that the emphasis in clinical settings is often on cognitive symptoms, an instrument such as this could provide an opportunity for a structured discussion around stigma and discrimination experiences important to people living with dementia, as well as planning psychosocial services and initiatives to reduce stigma and discrimination.

## Supporting information

Bhatt et al. supplementary material Bhatt et al. supplementary material

## Data Availability

The data that support the findings of this study are available from Alzheimer's Disease International. Restrictions apply to the availability of these data, which were used under licence for this study. Data are available from corresponding author, S.E.-L., with the permission of Alzheimer's Disease International.
