# Supplementary material for: The development and validation of the Discrimination and Stigma Scale Ultra Short for People Living with Dementia (DISCUS-Dementia)
Source: BJPsych Open. 2023 Aug 31;9(5):e164. doi: 10.1192/bjo.2023.551 (PMC10594093; doi:10.1192/bjo.2023.551)
Supplement: Bhatt et al. supplementary material [file S2056472423005513sup001.docx]

**Supplementary figure S1. Scree Plot**


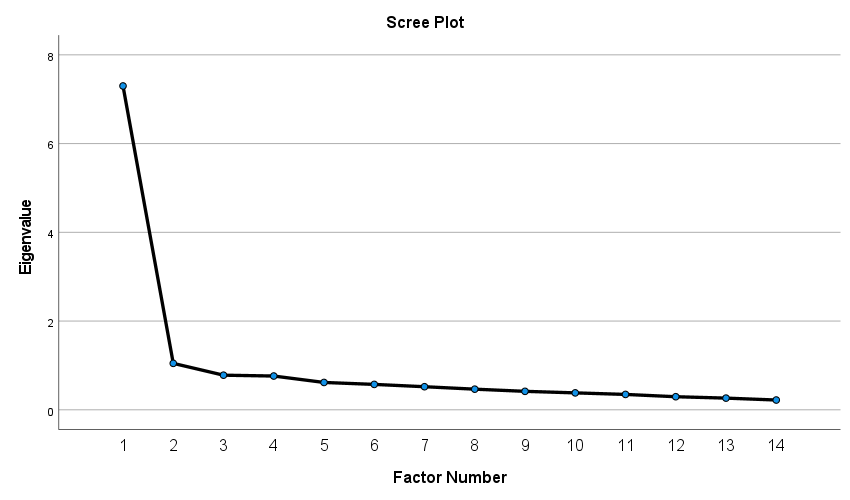


**Supplementary Table 1 Modification of the existing DISCUS Mental health scale items for use with people living with dementia based on stakeholder feedback**

| **Original DISC item** | **DISCUS-Dementia item (adaptations to original item in bold font)** |
| --- | --- |
| Have you been treated unfairly in dating or intimate relationships? | Have you been treated unfairly in dating or intimate relationships? |
| Have you been treated unfairly in making or keeping friends? | Have you been treated unfairly in making or keeping friends? |
| Have you been treated unfairly in housing? | Have you been treated unfairly in housing **(including having to move your house,** **move to a nursing home before you felt that you were ready or becoming homeless)?** |
| Have you been treated unfairly in your social life? | Have you been treated unfairly in your social life **(for example, being excluded from socialising, hobbies, attending events, leisure activities)?** |
| Have you been treated unfairly in your levels of privacy? | Have you been treated unfairly in your levels of privacy **(for example, privacy in hospital and / or in community settings, e.g. private letters or phone calls, medical records, criminal records bureau check)?** |
| Have you been treated unfairly in your personal safety and security? | Have you been treated unfairly in your personal safety and security? **(for example, verbal abuse, physical abuse, assault)** |
| Have you been treated unfairly in starting a family or having children? | **Have you been treated unfairly by your children or other family members ?** |
| Have you been avoided or shunned by people who know that you have a mental health problem | Have you been avoided or shunned by people who know that you have dementia **(for example, people do not want to come to your house or do not invite you out)**? |
| Have you been treated unfairly in finding a job? | REMOVED |
| Have you been treated unfairly in keeping a job? | REMOVED |
|  | **Have you been treated unfairly by health or medical staff (for example, did a health care professional suggest that you disengage from daily activities or social situation)^1^** |
|  | **Have you had rights or responsibilities unfairly taken away from you (for example, in family taking over financial decisions without a legal basis)?** |
|  | **Do people do things for you that you could do yourself because they know you have dementia?** |
|  | **Have you been told that you couldn’t do something that you still thought you could do?** |
|  | **Do people often joke about your dementia symptoms?** |
|  | **Because of your dementia have some people not taken your opinions seriously?** |

**^1^**Adapted from the original DISC-12: Brohan E, Clement S, Rose D, Sartorius N, Slade M, Thornicroft G. Development and psychometric evaluation of the discrimination and stigma scale (DISC). *Psychiatry Res*. 2013;208(1):33-40.

**Supplementary Table 2. Participant socio-demographic characteristics for total sample (n=704)**

| **Variable** | **N(%) or Mean(SD)** | | | **Variable** | **N(%)** | |
| --- | --- | --- | --- | --- | --- | --- |
| Sex | Male | | 281(37.7%) | Country | Argentina | 6(.8%) |
|  | /Female | | 464(62.3%) |  | Australia | 27(3.6%) |
| Age | N=745, Range: 24-94 | | 65.6(11.52) |  | Belgium | 10(1.3%) |
| Employment status | Full time paid employment | | 96(12.9%) |  | Brazil | 45(6.0%) |
|  | Part time paid employment | | 28(3.8%) |  | Canada | 29(3.9%) |
|  | Self-employed | | 48(6.4%) |  | China | 12(1.6%) |
|  | Unpaid/voluntary work | | 54(7.2%) |  | Colombia | 2(0.3%) |
|  | Unpaid carer | | 18(2.4%) |  | Costa Rica | 4(0.5%) |
|  | Retired | | 422(59.9%) |  | Croatia | 3(0.4%) |
|  | Student | | 4(0.5) |  | Dominican Republic | 1(0.1%) |
|  | Illness/sick-leave | | 44(5.9%) |  |  |  |
|  | Looking for paid work, unemployed | | 14(1.9%) |  | France | 2(0.3%) |
|  |  |  |  |  | Germany | 3(0.4%) |
| Education | Less than primary/elementary school | | 6(0.8%) |  | Greece | 6(0.8%) |
|  |  |  |  |  | Iceland | 1(0.1%) |
|  | Primary/elementary school | | 27(3.6%) |  | India | 14(1.9%) |
|  | Secondary school | | 67(9.0%) |  | Indonesia | 10(1.3%) |
|  | High school (or equivalent) | | 107(14.4%) |  | Iran | 1(0.1%) |
|  | Vocational training or apprenticeship | | 100(13.4%) |  | Ireland | 1(0.1%) |
|  |  |  |  |  | Italy | 50(6.7%) |
|  | College/pre-university/university | | 262(35.2%) |  | Japan | 8(1.1%) |
|  |  |  |  |  | Kenya | 1(0.1%) |
|  | Post graduate degree completed | | 176(23.6%) |  | Lebanon | 1(0.1%) |
| Area of residence | Urban | | 336(45.1%) |  | Malaysia | 10(1.3%) |
|  | Suburban | | 173(23.2%) |  | Mauritius | 1(0.1%) |
|  | Semi-rural | | 164(22.0%) |  | Mexico | 7(0.9%) |
|  | Rural | | 61(8.2%) |  | Netherlands | 208(27.9%) |
| Mean DISCUS-Dementia^2^ | n=720 | Range: 0, 2.79 | 0.44(0.46) |  | New Zealand | 17(2.3%) |
|  |  |  |  |  | Norway | 1(.1%) |
| Stigma impact scale | n=731 | Range: 0, 78 | 42.25(16.22) |  | Philippines | 2(.3%) |
|  |  |  |  |  | Portugal | 2(0.3%) |
| WEMWBS total | n=713 | Range: 15, 70 | 44.69(10.67) |  | Puerto Rico | 1(0.1%) |
| DEMQoL total | n=682 | Range: 1.10, 2.80 | 1.98(0.31) |  | Qatar | 3(0.4%) |
| WEMWBS categorical | Higher mental wellbeing ≥42 | | 435(58.4%) |  | Russia | 2(0.3%) |
|  | Lower mental wellbeing (0-41) | | 278(37.3%) |  | Singapore | 1(0.1%) |
| DQoL categorical | Higher QoL (> median 2.25) | | 110(14.8%) |  | Slovenia | 4(0.5%) |
|  | Lower QoL (< median 2.25) | | 348(76.8%) |  | South Africa | 5(0.7%) |
| WHO Region | African Region | | 7(0.9%) |  | Spain | 2(0.3%) |
|  | Eastern Mediterranean Region | | 5(0.7%) |  | Taiwan | 18(2.4%) |
|  | European Region | | 333(44.7%) |  | Thailand | 5(0.7%) |
|  | Region of the Americas | | 260(34.9%) |  | United Kingdom | 38(5.1%) |
|  | South-East Asia Region | | 29(3.9%) |  | United States | 165(22.1%) |
|  | Western Pacific Region | | 111(14.9%) |  | Vietnam | 16(2.1%) |
| World bank income categories | High-income economies | | 606(81.3) |  |  | |
|  | Upper-middle economies | | 96(12.9) |  |  |  |
|  | Lower-middle economies | | 43(5.8) |  |  |  |
